# China's Engagement with Global Health Diplomacy: Was SARS a Watershed?

**DOI:** 10.1371/journal.pmed.1000266

**Published:** 2010-04-27

**Authors:** Lai-Ha Chan, Lucy Chen, Jin Xu

**Affiliations:** 1 UTS China Research Centre, University of Technology, Sydney, Australia; 2 Institute for Global Health, Peking University, Beijing, China; 3 Health Science Center, Peking University, Beijing, China; London School of Hygiene & Tropical Medicine, United Kingdom

## Abstract

As part of the *PLoS Medicine* series on Global Health Diplomacy, Lai-Han Chan and colleagues provide a case study of China's growing engagement in global health diplomacy following the SARS epidemic.

*This article is part of the* PLoS Medicine *Global Health Diplomacy series.*

**Summary Points**

- SARS not only exposed a fundamental shortcoming of China's public health surveillance system as well as its single-minded pursuit of economic growth since the late 1970s, but also forced China to realize that, in the era of globalization, public health is no longer a domestic, social issue that can be isolated from foreign-policy concern.
- Its ailing health care system, its aspiration to be seen as a “responsible state,” and international demands for health cooperation have compelled China to be more proactive in the global health domain.
- There are signs that China is now using public health as a means to strengthen its diplomatic relations with the developing world, in particular the African continent.
- While China has embraced multilateral cooperation in a wide array of global health issues, its engagement remains “state centric” and therefore leaders attach primary significance to intergovernmental organizations, particularly the UN agencies.

## 

Severe acute respiratory syndrome (SARS) was the first global epidemic of the 21st century. It not only caused mass panic but also generated a discourse on health insecurity around the world. Table 1 shows a chronological account of the disease outbreaks. Owing to China's belated response, particularly its obstruction in early 2003 of the entry of World Health Organization (WHO) assessment teams into the country for investigation of the virus, the subsequent mapping of the disease during the outbreak period kept global attention on China. In retrospect, there appear to be valuable lessons China can draw from its experience with SARS and several implications of SARS on China's engagement in global health diplomacy. This case study examines China's policy changes in the area of public health since the SARS outbreak. Using literature reviews, personal experience, and informal interviews with Chinese health officials, we provide insight into the extent of China's increased engagement in public health, at both the domestic and the international levels.

**Table 1:** The chronology of the SARS outbreak.

Date	Major Events
16 November 2002	The first known case of SARS occurs in Foshan, Guangdong, southern China.
8 February 2003	Guangdong government informs the central government in Beijing about the outbreak.
11–14 February	Vice-mayor of Guangzhou says that the city is coping with the outbreak of atypical pneumonia and that “no extraordinary measures are needed.” On the 14th, the Ministry of Health officially tells WHO that the disease is under control in Guangdong.
21 February	Hong Kong index case arrives in the Metropole Hotel from Guangdong; the virus starts to spread globally.
11 March	The WHO Director-General, Gro Harlem Brundtland, raises member states' concern over the lack of information about the Guangdong outbreak to the WHO representative and asks him to convey it to Chinese Ministry of Health. On the same day, Hong Kong reports the Prince of Wales Hospital outbreak to WHO.
12–13 March	WHO issues global alert about atypical pneumonia; China's Health Minister accepts a WHO mission to examine the Guangdong outbreak.
15 March	WHO officially names the disease as “severe acute respiratory syndrome” (SARS).
17 March	China insists Guangdong's outbreak is “well under control.”
2 April	WHO issues travel advisory for Hong Kong and Guangdong province.
3 April	The Guangdong government allows a WHO team to investigate SARS in the province; Health Minister Zhang Wenkang states that China is “safe.”
8 April	Jiang Yanyong, a retired Chinese surgeon, exposes the underreporting of SARS cases in Beijing to *Time* magazine.
17 April	After a Politburo meeting, Beijing announces a national “war” on the virus.
20 April	Health Minister Zhang Wenkang and Beijing's Mayor Meng Xuenong were sacked for negligence in dealing with the disease. The Ministry of Health announces confirmed cases of SARS, which are at 9 times as the day before.
22 April	A major outbreak in Taiwan begins.
23 April	The SARS Control and Prevention Headquarters of the State Council is established with Vice-Premier Wu Yi as commander-in-chief; WHO issues travel advisory for Toronto, Beijing, and Shanxi province.
27 April	Xiaotangshan SARS hospital is completed in 8 days, involving 7,000 workers in Beijing.
29 April	Wen Jiabao takes part in an ASEAN-China Leaders' Meeting on SARS.
3 May	WHO sends three officials to Taiwan with Beijing's consent.
8 May	WHO issues travel advisory for Tianjin, Inner Mongolia, and Taipei.
14 May	WHO team meets Wu Yi.
15 May	The Chinese government passes a new law against those who break SARS quarantine and deliberately spread the disease.
23 May	WHO lifts travel advisory for Hong Kong and Guangdong.
27 May	Delegates to the World Health Assembly approve a resolution on SARS and revising the International Health Regulations.
5 July	WHO announces that SARS is under control worldwide.

From [39].

## China since the SARS Outbreak

We spoke with three high-ranking health officials in China's Ministry of Health in August 2009 who admitted that the SARS outbreak had alerted Chinese citizens as well as the government to the danger that public health, particularly infectious diseases, could become a dire threat if not properly controlled. This perceived threat extended beyond their country to the world. In the face of criticism from abroad about China's handling of the SARS epidemic, the new Hu Jintao–Wen Jiabao leadership, taking office in early 2003, swiftly adopted a more open and proactive attitude to the WHO member countries and southeast Asian nations containing the disease.

**Figure 1:**
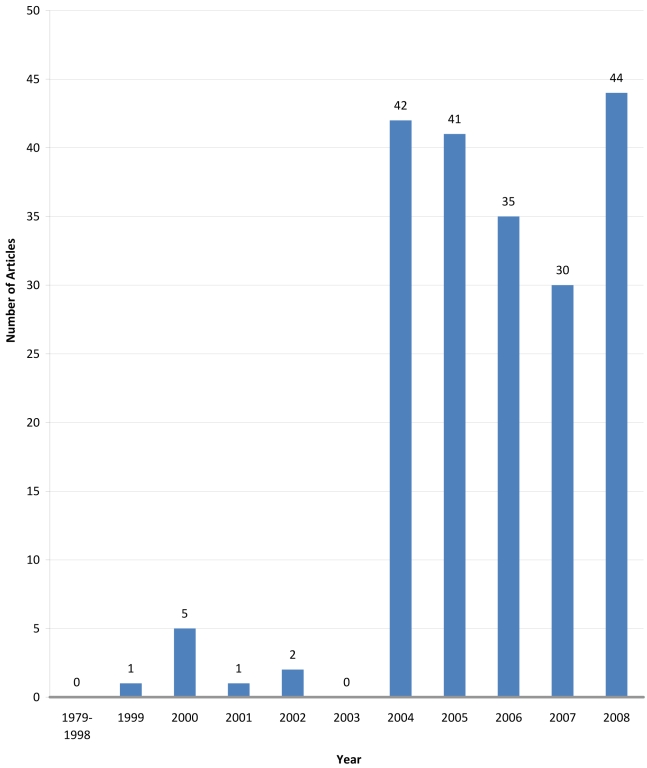
Number of Chinese journal articles per year on non-traditional security issues since 1979.

Indeed, SARS appears to have prompted a national discourse on the inter-relationship between infectious diseases and non-traditional security inside China. This is evidenced by the vast amount of literature on the subject of non-traditional security issues generated by Chinese scholars since the SARS outbreak. Using “
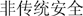
(*fei chuantong anquan*, non-traditional security)” to search for articles contained in a database known as “China Academic Journals Full-text Database: Economics, Politics and Law (Z*hongguo qikan quanwen shujuku: jingji, zhengzhi yu falü zhuandang, 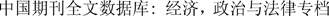
*),” there are barely any “non traditional security” articles published before the SARS outbreak. However, subsequent to the outbreak it became a flourishing subject in China's scholarly world. Among the articles that include “non-traditional security” in their titles since the start of economic reforms in 1979, more than 95% of them were published after 2003 (see Figure 1).

At the domestic level, the SARS outbreak exposed a fundamental shortcoming of China's health care system. As such, China required a national health reform in order to improve its surveillance system and reorient its single-minded pursuit of economic growth since the late 1970s to a more balanced development between economic growth and social infrastructure building [1]–[3]. The health officials in Beijing were also of the view that SARS could be seen as a turning point for China's health reform because it provided a political rationale for the government to accelerate the reform. According to the Asian Development Bank, SARS cost China US$6.1 billion, or 0.5% of its GDP, in 2003 [4]. This economic loss may seem insignificant, but for a regime that prioritizes economic growth and stability, the political repercussions of an economic decline caused by a health crisis cannot be underestimated. Indeed, SARS alerted the Chinese leadership to the pitfalls of a public health care system in disarray [2]. In order to maintain a sustained economic growth, the central government has increased its public health funding significantly since the SARS outbreak. For example, in 2003, the central and local governments altogether allocated 111.69 billion yuan for public health, an increase of 23% over the previous year. Between 2002 and 2006, the government's public health spending grew by almost 100%. There was a further increase of 29.1% in 2007 to 229.71 billion yuan. The share of public health spending in the country's GDP was 0.89% in 2007, compared to merely 0.75% five years ago [5],[6].

External pressure has also impacted on the development of China's public health. During the SARS outbreak, the WHO directly told the Chinese government in its mission report in April 2003 that “[t]here was an urgent need to improve surveillance and infection control” in the country [7]. Two years later, the Chinese government officially admitted its health care system was ailing in a joint report issued by State Council's Development Research Centre and the WHO [8]. The recent decision on a new rural cooperative medical system is one of its efforts to provide its rural residents by 2010 with more equitable and accessible health care [9]–[11] and improve its diseases surveillance system at the local level. In addition, both the “loss of face” in the SARS outbreak and its aspiration to be seen and respected as “a responsible state” have pushed China to enhance its cooperation with international institutions in dealing with other pressing health issues [12].

One of the prominent examples is the problem of HIV/AIDS. China is now working with multiple actors, including UN agencies (i.e., UNAIDS, WHO, UNICEF, the International Labour Organization, and World Bank), international non-governmental organizations (i.e., The Global Fund to Fight AIDS, Tuberculosis and Malaria, Bill & Melinda Gates Foundation, and Clinton Foundation), other states (i.e., United States, United Kingdom, and Australia), as well as non-governmental organizations inside the country to combat the disease. However, while Beijing calls for and welcomes involvement of multiple actors in combating the disease inside its territory, it maintains little tolerance of anyone or any activity that would attenuate its absolute control over the country or threaten the supreme authority of the government. A major feature of China's multilateral public health engagement, then, is that of “state-led health governance.”

Nevertheless, compared to its initial handling of SARS, China now reacts in more timely fashion in releasing information on contagious diseases, despite implementation problems that include sluggish responses to disease outbreaks on the part of local officials and technical incapacity to detect sudden outbreaks at the local level. In addition, China has shown increased willingness to engage with international organizations on a wide array of global health issues. For example, in order to align with international interests in tobacco control, Beijing signed the WHO Framework Convention on Tobacco Control (FCTC) treaty in December 2003. It was ratified in 2005 by the Chinese National People's Congress and its legislature and took effect in 2006 [13]. Being the largest producer as well as the largest consumer of tobacco in the world, Chinese tobacco policy has long been influenced more by economic concerns than by public health [14]. Its ratification of the FCTC came as a surprise for many.

China's recent responses to the 2009 outbreak of swine flu (influenza A/H1N1) give an impression that the dreadful effect of SARS six years ago has taught China a lesson. As soon as the WHO raised its pandemic alert phase from 3 to 4 on 28 April 2009, Premier Wen Jiabao convened a cabinet meeting to discuss a set of response measures designed to deal with the disease, although there was neither any reported case of swine flu in China at that time nor a similar virus found in the pigs in the country. Two days later, the Communist Party of China (CPC) General Secretary Hu Jintao convened a meeting of the Standing Committee of the Politburo. That the holding of the highest level meeting was announced immediately after its adjournment was regarded as unusual by many China watchers [15]. China's aggressive and visible approach towards swine flu appears to demonstrate the government's determination in tackling the disease. However, this aggressive or even excessively stringent measure against swine flu, as some observers have said, aroused international debate. The WHO asked China to justify its decision to keep travelers from Mexico in quarantine. The Mexican government criticized China's response as “unjustified,” threatened to take retaliatory action, and sent an airplane to Shanghai on 5 May 2009 to repatriate its quarantined citizens [16]–[18]. In contrast, others including health experts reportedly praised China for exercising extra vigilance against the virus [19],[20].

## A More Proactive Stance in Global Health Diplomacy

At the international level, there have been signs since the SARS outbreak that public health is high on China's foreign policy agenda. First, Beijing has become more proactive in participating in global health governance. China had for a long time played a passive role in the WHO since gaining its membership in the organization more than three decades ago. The SARS outbreak let China experience the power of the WHO, which has become increasingly more influential while other international organizations, such as the United Nations Security Council, International Monetary Fund, World Bank, G8, and the World Trade Organization, are facing legitimacy, accountability, and representativeness challenges. WHO's authority in dealing with disease outbreaks is still widely recognized [21]. Without China's prior consent, the WHO issued a travel advisory against unnecessary travel to Guangdong province, putting China under the global spotlight for spreading infectious disease to other countries. Perhaps this lesson has prompted the Chinese government to realize the political importance of the WHO and to increase its participation in global health governance.

In the WHO Director-General election in 2006, China, for the first time since it gained its membership in UN agencies in 1971, nominated and supported a Chinese national, Margaret Chan, as a candidate for the top post. It is widely believed that Chan's success was a diplomatic triumph both for her and for China. Wang Yizhou, then with the Chinese Academy of Social Sciences, Beijing, told one of the authors (LHC) in March 2008 that Margaret Chan's nomination as the Director-General of the WHO was not a fortuitous incident. The health officials we spoke with in Beijing concurred with Wang's view and explained that China has recently realized and valued the increasing importance of the WHO at the world stage. It is also a source of national pride to have a Chinese national at the top post of the global health organization [22]. Chan was the Director of Health of Hong Kong during the SARS outbreak in 2003. Her nomination could be seen as a case of China's smart play and rising clout at the global stage, displaying its confidence in her managing of Hong Kong affairs and the successful implementation of China's “One Country, Two Systems” policy [23].

Furthermore, China's WHO role politically could be regarded as a pre-emptive measure to block Taiwan's attempts to seek WHO membership [23]. On the other hand, with improved relations with the Ma Ying-jeou administration in Taiwan, China has become more flexible in seeking cross-strait cooperation in health. For example, as a consultant for the Chinese Medical Association, Chinese Vice Minister of Health Huang Jiefu attended a conference on “Cross-Strait Cooperation in Preventing H1N1” in Taiwan in January 2010. During the meeting, Huang emphasized an extensive cross-strait collaboration in the area of public health, including disease notification and food safety [24]. In addition, Beijing dropped in 2009 its objection to Taiwan's application for an observer in the World Health Assembly. That being said, Taiwan's participation is allegedly required to be in line with Beijing's “One China” policy [25].

## More Public Health Assistance and Diplomacy

The second sign that China has put public health high on their foreign policy agenda since SARS is their provision of development assistance and global public goods for health. As such, China is now using public health as a means to strengthen its diplomatic relations with the developing world, including African countries. China began in the 1960s to send “angels in white” and “barefoot doctors” to the sub-Saharan region to provide some of the poorest African countries with medical services. However, as argued by Huang Yanzhong of Seton Hall University, China's health diplomacy was “flimsy, passive, and asymmetric,” at least until the 1980s [3]. After the SARS outbreak, in spite of its own failing health system, the Chinese government reiterated in its China's African Policy, published in early 2006, the nation's commitment to improving Africa's public health service.

To balance the criticisms that its energy and resource extraction in Africa grab the scarce resources there and that it shields disreputable regimes in such countries as Sudan and Zimbabwe from international opprobrium, China has stressed “win-win” relations in its deepening engagement with African countries. In response to the claims of exploitation in the natural resource sectors [26],[27], China emphasizes a no-strings-attached policy in offering financial aid and technical support to less developed countries, including those in the African continent. In contrast, donor countries in the West and international financial institutions often attach conditionalities to their foreign aid programs, which are linked to market and political liberalization and good governance [28]. China has expanded its public health initiatives, such as in infrastructural building and health practitioner training, in Africa in recent years [29], as well as commitment to cooperation with many African countries to help prevent and treat infectious diseases, particularly HIV/AIDS and malaria [30]–[34]. In his African visit in June 2006, Chinese Premier Wen Jiabao asserted that China would promote sustainable development and help African countries tackle their burning social problems, of which public health was one of the top priorities [35]. Again in November 2009, during the fourth ministerial meeting of the Forum on China-Africa Cooperation in Egypt, Wen announced eight new measures to strengthen China-Africa cooperation in the following three years, including a 500 million yuan (US$73.2 million) assistance package that allows China to build 30 hospitals and 30 malaria prevention and treatment centers and to train 3,000 practitioners in the continent [36].

Undoubtedly China has been learning from itself as well as from other developed countries the importance of providing sustainable development and global public goods for improving one's reputation on the world stage. At the “First International Roundtable on China-African Health Collaboration – New Health Initiative” in December 2009 in Beijing, one of us (LC) observed representatives from the WHO, World Bank, and the Bill & Melinda Gates Foundation praising China's development packages for their positive contributions to African development.

In addition, China's State Council has established in recent years a coordinating mechanism to facilitate cross-ministry dialogues and cooperation in global health and foreign aid initiatives. Chinese scholars have noted that a State Council “Global Health Diplomatic Coordination Office” (*quanqiu weisheng waijiao xietiao bangongshi*, 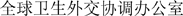), led by a senior official at vice-Premier level, is crucial to effectively coordinating and developing policies of health diplomacy [37]. In order to increase the capacity of China's health diplomats to deal with global health challenges, a training course, the first in a series, for Chinese officials, including officials from the Ministries of Foreign Affairs and Health, was held in August 2009 in the Institute for Global Health at Peking University [38].

## Was SARS a Watershed?

Following the Chinese government's acknowledgement of a SARS outbreak in the country, it began to acknowledge the importance of public health to national development and to accordingly strengthen its multilateral cooperation in combating contagious diseases inside and beyond its borders. For example, in the midst of the recent global economic downturn, the Chinese government announced in 2009 an injection of 850 billion yuan (US$125 billion) into its health care system to improve its operation. Since the SARS outbreak, it has not only deepened its engagement with other nations and international organizations, and cooperated with a variety of actors in dealing with its own fledgling health care system including the problem of HIV/AIDS, but China has also developed a vision for global health diplomacy. A ground-breaking implication of the SARS outbreak for China is that it was struck to realize that public health is not simply a domestic, social issue that can be isolated from foreign-policy and security concerns. In a globalizing world, the Chinese government appears to have learned that its health policy will be scrutinized by the world, and hence, it has become more open to and actively participates in global health governance. The government is now learning from such European countries as the UK, France, and Switzerland in the provision of the global public goods for health. Its substantial health assistance to sub-Saharan Africa in building hospitals and training health practitioners forms part of its health diplomacy and contribution to global health governance. It has also been proactively engaging with both regional and global health institutions since 2003 and set up different health surveillance networks with its ASEAN partners as well as other intergovernmental organizations, such as the Asia-Pacific Economic Cooperation (APEC) forum [12].

Despite its increasing engagement with global health governance since the SARS outbreak, China's approach remains, however, fundamentally *state-centric*, contrary to the essence of *global* health diplomacy and governance. With grave concern about the loss of national sovereignty to external or nongovernmental actors, Chinese senior leaders have therefore attached primary significance to intergovernmental organizations, particularly the UN agencies. In evaluating the impact of SARS, Andrew Price-Smith has put the same point succinctly: “while the SARS epidemic may have generated moderate institutional change at the domestic level …, it resulted in only ephemeral change at the level of global governance” [2]. In other words, national sovereignty is still of paramount importance for the Chinese leadership. Because of its sensitivity to foreign interference into its internal affairs, the Chinese government has not yet formally or officially endorsed the notion of “human security.” Under the umbrella concept of national security, “human safety,” instead of “human security,” is discussed throughout all of China's five white papers on national defense since 2000 (i.e., 2000, 2002, 2004, 2006, and 2008). Taiwan's participation in the World Health Assembly is predicated on the condition that it is considered part of China, not an independent entity. Having no tolerance in ceding its supreme authority, the central government has adopted a multi-faceted attitude towards its civil society organizations. While Beijing shows its willingness to cooperate with a wide array of actors inside China, it refuses to let its domestic NGOs and activists establish direct links with their counterparts overseas.

It is still uncertain whether this sovereign concern will trump the provision of global public good for health. Nevertheless, in a highly globalizing world, infectious diseases know no border. While China is seeking to adhere as much as possible to the underlying norms and rules of global health governance (and sometimes even applies them to their extremes), as evidenced by its handling of the recent swine flu outbreak, the major step forward is perhaps to reframe health as a *global* public good that is available to each and every individual of the world, rather than merely as an issue of concern to nation-states.

## Abbreviations

FCTC: Framework Convention on Tobacco Control
SARS: severe acute respiratory syndrome
WHO: World Health Organization

## References

[1] Gu X, Wong J, Zheng YN (2004). Healthcare regime change and the SARS outbreak in China.. The SARS epidemic: challenges to China's crisis management.

[2] Price-Smith AT (2009). Contagion and chaos: disease, ecology, and national security in the era of globalization.

[3] Huang YZ, Freeman CW, Lu XQ (March 2009). China's new health diplomacy.. China's capacity to manage infectious diseases: global implications.

[4] (15–16 December 2003). Speech by Dr Henk Bekedam, WHO representative in China at the International Forum on SARS Prevention and Control.. http://www.wpro.who.int/china/media_centre/speeches/speech_20031215.htm.

[5] Zhongguo tongji nianjian (China Statistical Yearbook).

[6] (2005). Zhongguo weisheng nianjian (Yearbook of Public Health in China).

[7] Schnur A, Kleinman A, Watson JL (2006). The role of the WHO in combating SARS, focusing on the efforts in China.. SARS in China: prelude to pandemic?.

[8] (30 July 2005). Medical reform ‘basically unsuccessful’.. http://www.chinadaily.com.cn/english/doc/2005-07/30/content_464795.htm.

[9] Wang SG (July 2009). Adapting by learning: the evolution of China's rural health care financing.. Mod China.

[10] Yip W, Hsiao WC (2009). Non-evidence-based policy: how effective is China's new cooperative medical scheme in reducing medical impoverishment?. Social Science & Medicine.

[11] Brown PH, Brauw de A, Du Y (June 2009). Understanding variation in the design of China's new co-operative medical system.. China Q.

[12] Chan LH, Lee PK, Chan G (January 2009). China engages global health governance: processes and dilemmas.. Global Public Health.

[13] (29 August 2005). China ratifies WHO convention on tobacco control.. http://www.highbeam.com/doc/1G1-135593920.html.

[14] Hu T-W, Mao Z, Ong M, Tong E, Tao M (2006). China at the crossroads: the economics of tobacco and health.. Tob Control.

[15] Bradsher K (1 May 2009). China's leaders take visible approach to swine flu.

[16] Bezlova A (5 May 2009). China swine flu response criticised as ‘unjustified’.

[17] Anderilini J, Jack A (4 May 2009). Mexico hits at China's quarantine policy.

[18] Jack A, Hille K, Thomson A (5 May 2009). WHO tackles China on swine flu measures.

[19] Cha AE (29 May 2009). Caught in China's aggressive swine flu net.

[20] Wong E (11 November 2009). China's tough flu measures appear to be effective.

[21] Chan LH (January 2010). WHO – the world's most powerful international Organizations?. J Epidemiol Community Health.

[22] Chan WY, Ma SY (2009). The making of a Chinese head of the WHO: a study of the media discourse on Margaret Chan's contest for the WHO Director-Generalship and its implications for the collective memory of SARS.. Int J Health Serv.

[23] Shen S (Fall 2008). Borrowing the Hong Kong identity for Chinese diplomacy: implications of Margaret Chan's World Health Organization election campaign.. Pac Aff.

[24] (20 January 2010). Cross-strait talks agenda urged to include healthcare.. http://www.taiwantoday.tw/ct.asp?xItem=92376&CtNode=413.

[25] (30 April 2009). A healthy development.

[26] Brooks P (9 February 2007). Into Africa: China's grab for influence and oil.. Heritage Lectures.

[27] Klare M (2008). Rising powers, shrinking planet: how scare energy is creating a new world order.

[28] Smith BC (2007). Good governance and development.

[29] Morrison JS (4 June 2008). China in Africa: implications for US policy.

[30] Ministry of Foreign Affairs of the PRC (January 2006). China's African policy.

[31] (1 June 2005). Guochan Aiziyao chukou Nanfei (Chinese-made AIDS drug exports to South Africa).

[32] (18 January 2007). Chinese scientists take malaria fight to Africa.. http://www.alertnet.org/thenews/newsdesk/B155919.htm.

[33] Lague D (5 June 2007). On island off Africa, China tries to wipe out malaria.

[34] Tan EL (5 November 2009). China adopts ‘malaria diplomacy’ as part of Africa push.. http://www.reuters.com/article/idUSSP503140.

[35] Stamp G (26 June 2006). China defends its African relations.. http://news.bbc.co.uk/2/hi/business/5114980.stm.

[36] (9 November 2009). Chinese premier announces eight new measures to enhance cooperation with Africa.. http://english.peopledaily.com.cn/90001/90776/90883/6807055.html.

[37] Chen L, Xu WZ (2009). Bianhua de Feizhou xuyao woguo xinxing de waisheng waijiao celüe (The changing Africa necessitates our country's new health diplomatic strategies).

[38] Ministry of Health (12 August 2009). Quanqiu weisheng waijiao peixunban zai jing juban (Global health diplomacy training course held in Beijing).. http://www.moh.gov.cn/publicfiles/business/htmlfiles/mohgjhzs/s7952/200908/42331.htm.

[39] World Health Organization (2006). SARS: how a global epidemic was stopped.

